# Evaluation of efficacy and safety of rivaroxaban combined with aspirin in patients with chronic coronary artery disease: A protocol for systematic review and meta-analysis

**DOI:** 10.1097/MD.0000000000028779

**Published:** 2022-02-11

**Authors:** Han Wang, Xiuqi Li, Xingan Wu, Huiting Wu, Gang Xie, Hui Wang

**Affiliations:** a Department of Cardiology, General Hospital of the Yangtze River Shipping, Wuhan, Hubei, China; b Department of Cardiology, Caidian District People's Hospital, Union Jiangbei Hospital, Huazhong University of Science and Technology, Wuhan, Hubei, China.

**Keywords:** aspirin, coronary artery disease, efficacy, rivaroxaban, safety

## Abstract

**Background:**

Coronary artery disease (CAD) is among the main causes of morbidities and mortalities globally. It is also considered to be an outcome of acute thrombotic events which entail activating platelets as well as coagulation proteins. In particular, rivaroxaban along with aspirin have been considered to reduce thrombotic events. However, they are yet to be evaluated by combining with or putting them against each other in patients experiencing CAD. This study intends to carry out an evaluation of whether combining rivaroxaben with aspirin will be effective and safe in treating patients experiencing chronic CAD.

**Methods and analysis:**

We intend to search information from the following databases: MEDLINE, EMBASE, Web of Science, Cochrane library, WanFang, and China National Knowledge Infrastructure. In the search, we intend to regard randomized control trials written in either English or Chinese and only those published until December 2021, as well as only those that have assessed the effectiveness and safety of combining rivaroxaban and aspirin in treating patients suffering from chronic CAD. We intend to accompany the study identification with searching or relevant reference lists as well as citations. We will also contact respective authors to provide additional information or data were needful. From the search, we will collate all citations identified and remove all duplicates. Similarly, 2 independent authors will screen all the titles and abstracts and assess them against the inclusion criteria for the study. Only selected studies will be included for critical appraisal, extraction of data, and synthesis. We will then conduct statistical analyses by utilizing a random-effect model.

**Ethics and dissemination:**

This study does not require ethical approval as the findings will be published in a peer-review journal.

**Registration number:**

10.17605/OSF.IO/WBGE4

## 1 Introduction

Today, coronary artery disease (CAD) has become a worldwide medical condition as well as a prominent basis of morbidities and mortalities.^[1]^ In most cases, patients experiencing CAD appear to be at higher risks for conditions such as cardiovascular deaths, ischemic stroke, and myocardial infarctions (MIs). However, the fundamental pathophysiology of these specific events, especially in patients experiencing atherosclerosis includes the rapture or erosion of an atherosclerotic plaque, exposing the sub-endothelial matrix to the circulating blood.^[2]^ Subsequently, this causes an activation of platelet accumulation and clotting cascade. Accordingly, it can possibly cause an occlusive thrombus in the artery.^[3]^ Using aspirin is crucial to irreversibly block the thromboxane A2 from forming A2; thus, it is critical in reducing aggregation of platelets. In essence, aspirin is broadly used in preventing ischemic events in patients suffering from coronary heart complications. In essence, randomized trials have demonstrated that it contributes to about 20% risk reduction of vascular events.^[3]^

Meanwhile, using vitamin K antagonists, including warfarin can help in inhibiting or preventing the vitamin-dependent coagulation proteins’ functions, which is essential in also preventing thrombin from forming. It means that vitamin K antagonists can also contribute to lowering of cardiovascular events following MI. However, using them is still restricted by some possibilities for excessive bleeding.^[4]^ Some studies have assessed possibilities of a combined therapy with vitamin K antagonists and aspirin, with additional benefits against recurrent MI and death unlike using aspirin alone. Nevertheless, its clinical uptake is still limited due to potential serious bleeding, which can lead to intracranial hemorrhage.^[4]^ In particular, using factor Xa inhibitors can essentially offer more distinct competitive prevention of coagulation proteins with enhanced or same effectiveness to warfarin, as well as lowered rates of intracranial bleeding.^[5]^

Rivaroxaban is an oral factor Xa inhibitor, efficient in the treatment of intravenous thromboembolism. Over the years, using rivaroxaban has established that it helps in inhibiting thromboembolic events, especially in atrial fibrillation.^[6,7]^ Accordingly, it provides possible benefits over vitamin K antagonists. For example, rivaroxaban provides faster onset and offset action and involves fewer medication interactions.^[8]^ At the same time, rivaroxaban has a dual-mode excretion process. In particular, an estimated two-thirds of the drug can be eliminated by the liver through metabolism to an inactive metabolite. Similarly, a third of it can be removed unchanged via the kidney. Thus, rivaroxaban can be said to be fairly safer for patients, mainly for patients with diabetes as their conditions are often accompanied by renal dysfunction.^[9]^ To this end, using rivaroxaban and aspirin have confirmed the capacity to reduce thrombotic events, although this is yet to be tested using patients suffering from CAD. Thus, this study aims to explore the effectiveness and safety of combining rivaroxaban with aspirin to treat patients experiencing CAD.

## 2 Objective

The present study aims at investigating the possible effectiveness and safety of using rivaroxaban alongside aspirin to treat patients experiencing chronic CAD.

## 3 Methods and design

This study will be reported on the basis of the Preferred Reporting Items for Systematic Review and Meta-Analysis Protocols statement.^[10]^ The protocol is registered in the Open Science Framework (OSF, https://osf.io/).

### 3.1 Eligibility criteria

#### 3.1.1 Type of studies

We will include all randomized control trials (RCTs) along with quasi-RCTs. However, we will exclude all trials with no corresponding associations with the control group.

#### 3.1.2 Type of participants

We will also include all patients found to have CAD, meaning that we will not limit patients in terms of their age, ethnicities, and others.

#### 3.1.3 Type of interventions

The RCTs that include rivaroxaban combined with aspirin as the treatment will be included, as long as the RCTs including rivaroxaban alone or aspirin alone or other treatments.

#### 3.1.4 Outcome measures

The composite endpoint of death from any of the causes (MI or cardiovascular) was considered as the primary outcome. The secondary endpoint included mortalities, risks of MI, and risks of stroke, among others

### 3.2 Search methods

We searched data from the following databases: MEDLINE, EMBASE, Web of Science, Cochrane library, WanFang, and China National Knowledge Infrastructure. The search mainly regarded randomized control trials written in Chinese and English languages from their inception to December 2021, mainly those that assessed the effectiveness and safety of rivaroxaban combined with aspirin in patients with chronic CAD. The terms used in the search will be: “rivaroxaban ∗”, “aspirin∗”, “coronary artery disease”, “coronary heart disease”, “randomized control trials” as topic.

### 3.3 Data collection and analysis

#### 3.3.1 Study selection and data management

After we implemented the strategy for searching information from the selected databases, the authors imported all pinpointed referenced into the Endnote V9.0 software (Build 12062) and removed all duplicates. Accordingly, the 2 independent authors evaluated the studies identified in terms of their titles and abstracts for inclusion. They considered excluding irrelevant studies. Then, 1 author searched and retrieved all full text studies. At the same time, the authors assessed the retrieved full studies to ascertain whether they fit final inclusion. In case of disagreements between the authors, they will resolve them through a consensus. Studies with largest sample sizes will be selected where they are published in more than a single report. Besides, studies without full texts will be excluded.

#### 3.3.2 Data extraction

The author extracted data from every eligible study by establishing baseline participants’ features, types of interventions, nature of comparisons, outcomes, as well as duration of follow-up. The end point of the study included effectiveness and safety outcome. The authors addressed discrepancies and disagreements by involving a third author.

#### 3.3.3 Quality assessment and risk of bias

The studies’ quality was assessed by the 2 independent authors. They used a “risk of bias” tool as per the RevMan to assess the quality through utilization of sources of bias, including generation of sequence, concealment allocation, incomplete data, participants’ blinding, assessing outcomes, and selective reporting. Every study's methodology was grades as either “high”, “low”, or “unclear”, in order to establish the risk of bias.

#### 3.3.4 Measures of treatment effect

The authors expressed the effect sizes as either odds ratios or relative rations - for noncontinuous data. They also calculate the 95% of weighted or final post-intervention mean differences - for continuous data.

#### 3.3.5 Dealing with missing data

The authors will contact relevant author where there are missing data.

#### 3.3.6 Assessment of heterogeneity

We will test heterogeneity of results using different studies prior to merging the statistics. The study will also involve an evaluation of heterogeneity by means of *I*^*2*^ index. Where the *I*^*2*^ value is <50%, we will consider a non-substantial level of heterogeneity; hence, we will apply a fixed-effect model to the meta-analysis. However, we will use a random-effects model where the *I*^*2*^ value will be >50%, which is an indication of a significant heterogeneity.

#### 3.3.7 Assessment of reporting biases

We will investigate the bias of publication by utilizing the funnel plots. We will also employ the Egger regression test to statistics in case the funnel plots demonstrate asymmetry.

## 4 Discussion

This review aimed at assessing the effectiveness and safety of combining rivaroxaban with aspirin to treat patients experiencing CAD. Systematic reviews examining safety and effectiveness of rivaroxaban combined with aspirin to treat patients suffering from CAD have already been published; however, the results remain controversial. To this end, this study employs a systematic review and meta-analysis to assess rivaroxaban and aspirin's effectiveness and safety in treating patients experiencing CAD. Accordingly, these results demonstrate that there is a potential ranking for using rivaroxaban combined with aspirin in treating patients experiencing CAD.

## Author contributions

**Conceptualization:** Han Wang.

**Data curation:** Han Wang, Huiting Wu.

**Formal analysis:** Han Wang, Xingan Wu.

**Funding acquisition:** Xiuqi Li, Huiting Wu, Gang Xie, Hui Wang.

**Investigation:** Han Wang, Xiuqi Li, Xingan Wu.

**Methodology:** Huiting Wu, Gang Xie, Hui Wang.

**Project administration:** Han Wang, Xiuqi Li, Hui Wang.

**Resources:** Xingan Wu, Gang Xie, Hui Wang.

**Software:** Han Wang.

**Supervision:** Han Wang, Xiuqi Li, Huiting Wu, Hui Wang.

**Validation:** Han Wang, Xingan Wu, Gang Xie.

**Visualization:** Hui Wang.

**Writing – original draft:** Han Wang.

**Writing – review & editing:** Hui Wang.
